# Elbow flexion enables rescuers with low BMI to deliver chest compressions in compliance with CPR guideline recommendations

**DOI:** 10.1038/s41598-026-39671-5

**Published:** 2026-02-18

**Authors:** Katherine Thurlow, Lucas Rehnberg, Jelena Ivetić, Thais Russomano, Snezana Levic

**Affiliations:** 1 Brighton and Sussex Medical School, University of Sussex, Brighton, BN1 9PX UK; 2 General Intensive Care Unit, University Hospital Southampton, Tremona Road, Southampton, UK; 3 InnovaSpace, London, SE28 0LZ UK; 4 Faculty of Technical Sciences, University of Novi Sad, Novi Sad, Serbia; 5 Space Technology Center, AGH University of Krakow, Kraków, Poland; 6 School of Medicine, CEMA, University of Lisbon, Lisbon, Portugal; 7 Sensory Neuroscience Research Group, School of Pharmacy and Biomolecular Sciences, University of Brighton, Huxley Building, Brighton, BN2 4GJ UK

**Keywords:** Cardiopulmonary resuscitation, Hypogravity, BMI, Elbow flexion, Physiology, Cardiology, Medical research

## Abstract

High quality cardiopulmonary resuscitation (CPR) increases survival outcomes. Smaller rescuers have been found to be at risk of providing inadequate CPR, particularly relating to chest compression depth, especially in novice rescuers. This study aims to look at the quality of CPR provided by smaller rescuers, and to investigate any potential compensation techniques used such as elbow flexion and extension, to maintain adequate quality CPR. Healthy adult participants performed three five-minute sequences of CPR on a mannequin with springs of 3 different strengths, in a randomized order. An electrogoniometer attached to the elbow measured the flexion and extension throughout. The results suggest that chest compressions were maintained at recommended depth and rate levels despite the increase in spring stiffness by using elbow flexion and extension, especially in participants with lower BMI and increased spring stiffness. These findings suggest potential compensatory mechanisms that can be used to maintain good CPR in situations of the rescuer being significantly smaller than the patient, similarly to as has been suggested when delivering CPR in hypogravity, thus transferring knowledge from these environments to Earth. Using elbow flexion and extension should be taken into consideration when revising the internationally recognized CPR guidelines.

## Introduction

Cardio-pulmonary resuscitation (CPR) is a fundamental component of basic/advanced life support (BLS/ALS) and the chain of survival. Since its introduction in the 1960s, modern CPR with manual chest compressions at its foundation has been shown to improve survival following cardiac arrest^1^. It comprises of either continuous chest compressions alone or chest compressions and rescue breaths^2^. The chest compressions help to pump blood around the body, maintaining perfusion of the vital organs^1,2^.

Terrestrial CPR continues to be extensively researched, with international guidelines published every 5 years by the International Liaison Committee on Resuscitation [ILCOR]. In the United Kingdom (UK), approximately 55 per 100,000 people per year will have an out-of-hospital cardiac arrest and may require CPR^1^. Within hospitals, around 1 to 1.5 per 1000 admitted patients per year will experience an in-hospital cardiac arrest and may require CPR^1^. Rapid recognition and prompt action gives the patient the best chance of survival, as such, CPR is taught to healthcare professionals and laypersons worldwide^2^.

In recent years, there has been growing interest in the delivery of critical care medicine, including CPR, in extreme environments^3^, such as during spaceflight, particularly in light of the Artemis missions targeting the Moon and Mars^4^. The culmination of microgravity simulation studies led to publication of CPR guidelines in 2020 specifically for microgravity and in 2022 for hypogravity^5,6^.

Hypogravity and microgravity studies, where there is a reduction rather than a complete absence of gravitational field, like on the Moon and Mars, have shown an increase in the elbow flexion angle while performing CPR, compensating for the reduction in body weight^5^. In hypogravity, an elbow flexion angle of 14 ± 8 degrees has been shown to achieve adequate CPR performance^6,7^.

This finding contrasts with the traditional teaching of CPR on Earth, which advocates interlocked hands positioned on the lower half of the sternum, around the centre of the chest, and straight arms compressing the chest by approximately 5–6 cm twice per second^2^, where the rescuer accelerates their upper body toward the patient, transferring that force via their arms to compress the chest to the appropriate depth. However, smaller rescuers have been found to be at risk of providing inadequate CPR, particularly relating to chest compression depth, especially in novice rescuers, such as those performing out-of-hospital CPR^8,9^.

Despite these observations, it remains unclear whether similar compensatory mechanisms to those described in hypogravity are used, or could be beneficial, during terrestrial CPR when the rescuer is significantly smaller than the patient. The size difference between patient and rescuer may be apparent in several situations. Obesity is a factor in more than 5% of hospital admissions and is associated with an increased risk of cardiac arrest due to cardiovascular disease (CVD), heart failure, arrhythmia, and even sudden cardiac death^10,11^. The majority of nurses in the UK are female, so there is a likelihood that they may be smaller than their patients^12^. The Resuscitation Council UK states that CPR training forms part of the Health Education curriculum in schools in England for children aged 12 years and older. This inclusion is recommended as it is estimated that approximately 80% of cardiac arrests in the UK occur in the home^13^. A child providing CPR is therefore also likely to be a smaller rescuer than the patient. Similar to conditions in a reduced gravitational field, a rescuer with lower body weight could struggle to generate enough force through upper-body acceleration to compress the patient’s chest effectively using the traditional CPR technique^8,9^.

Therefore, the aim of this study was to evaluate whether elbow flexion and extension are used as a compensatory strategy to maintain CPR performance in accordance with current guidelines^2^, when the rescuer is of smaller size or performing CPR on a larger patient, using a terrestrial manikin model with variable chest stiffness.

## Methods

This study was conducted between September 2021 and March 2022 and approved by the Brighton and Sussex Medical School Research Governance and Ethics Committee (RGEC) (ER/BSMS6662/1, year of approval 2021). All research was performed in accordance with relevant guidelines/regulations.

### Sample size calculation

Sample size was estimated using two different approaches, corresponding to the two types of analyses used in the study. First, for group comparisons based on BMI or sex, we used an online calculator for independent samples t-tests (https://www.danielsoper.com/statcalc/calculator.aspx?id=47), with the following parameters: anticipated effect size (Cohen’s d) = 0.8 (large), statistical power = 0.80, and significance level α = 0.05, one-tailed. This yielded a recommended minimum of 42 participants in total, or 21 per group. Second, for repeated measures comparisons across spring stiffness conditions (three levels per participant), we estimated the required sample size for Friedman ANOVA using the G*Power software (version 3.1.9.7)^14^. Approximating the Friedman test via repeated measures ANOVA (within factors), and assuming a medium effect size (f = 0.25), α = 0.05, power = 0.80, and three repeated measures, the minimum sample size was calculated to be between 19 and 24 participants, depending on expected correlation among measures (ρ = 0.3–0.5).

Our study included 23 participants, which satisfies the estimated requirement for the repeated-measures comparisons. While this sample does not meet the calculated minimum of 42 participants for independent-group comparisons (e.g., by BMI or sex), these comparisons represent secondary aims of the study and were interpreted descriptively and exploratorily. The primary goal was to evaluate within-subject changes across spring stiffness levels and the influence of elbow flexion dynamics on CPR performance, for which the sample size was adequate. Additionally, for multivariate modeling, the full dataset of 69 observations (3 measurements per participant) was used.

### Participants

**Fig. 1:**
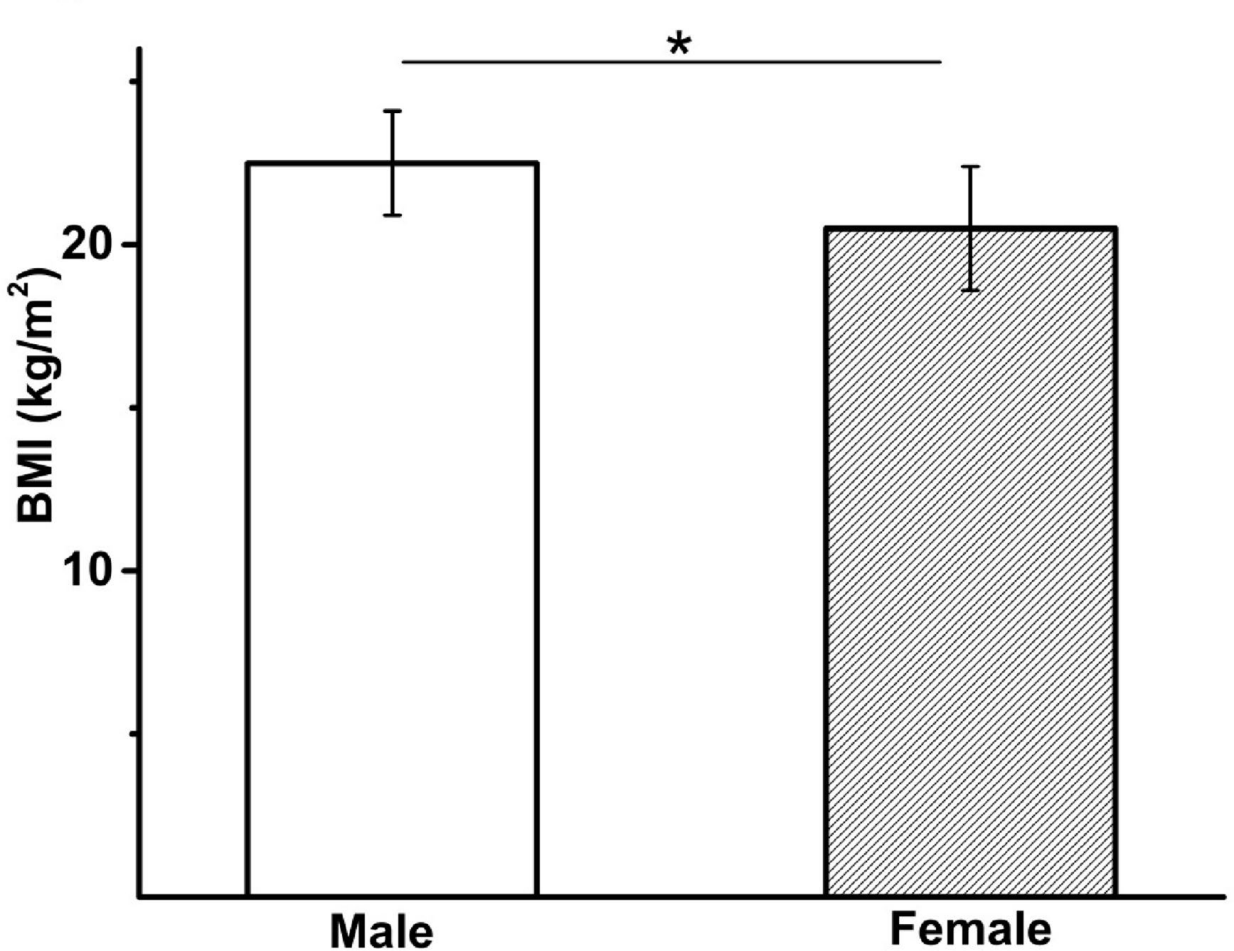
Comparison of BMI (kg/m^2^) reveals significant difference between male and female participants. Average BMI (kg/m^2^): male 22.5 ± 1.6 and female 20.5 ± 1.9, *p* = 0.015, male *n* = 9, female, *n* = 14. *Significance at *p* < 0.05.

This study recruited 23 healthy participants (university students) aged between 20-35years both male (*n* = 9) and female (*n* = 14) (Fig. 1). Healthy participants were recruited from the University of Sussex, UK, where all experiments were conducted. Participants received standard CPR training from a qualified instructor, according to Resuscitation Council UK guidelines^2^. Inclusion criteria required participants to be within the 20–35 year age range (typical university age population).

Exclusion criteria included any current or recent (< 1 week prior) viral illness, any significant chronic disease that might affect physical activity or elbow flexion/extension, and any regular medication use other than the contraceptive pill, to reduce the risk of confounding factors related to disease. Participants were selected based on their body mass index (BMI; normal or below normal, Fig. 1) and chest stiffness was used to assess the degree of elbow flexion needed to maintain adequate chest compression. Patient size was represented through BMI, and chest stiffness was adjusted to simulate varying levels of compression difficulty, associated with different BMIs. Parameters directly indicative of chest stiffness, such as mean chest compression depth (mm), were used to relate performance to participant size as determined by BMI.

### Equipment used

CPR was performed on a Laerdal adult BLS manikin with three different compression springs: a standard spring requiring 50 kg of force to compress to 50 mm (stiffness 9.8 N/m), an easy spring requiring 30 kg of force to compress to 50 mm (stiffness 5.88 N/m), and a hard spring requiring 60 kg of force to compress to 50 mm (stiffness 11.76 N/m). The manikin was equipped with digital CPR efficiency monitoring. An electrogoniometer (MBient Lab, San Francisco, CA, USA) was used to measure the angle of elbow flexion and extension during CPR performance. A pulse oximeter was used to measure the resting heart rate of participants, post-CPR heart rate, and to confirm that the heart rate had returned to baseline before starting the next CPR sequence.

### Experimental procedure

A standardized protocol was used for all participants. Following CPR training and the signing of informed consent, the height and weight of each participant was measured and BMI calculated. Resting heart rate was measured using pulse oximetry both before and after each CPR session. An electrogoniometer was attached to the elbow of the dominant arm and calibrated prior to each CPR performance.

The order of compression springs was randomized using software for random number generation (range 1–3), and the chosen spring was inserted into the manikin. Participants then performed 5 min of CPR following a pattern of 30 compressions followed by a 6-second rest period, simulating the time required for rescue breaths (30:2 ratio), although rescue breaths were not performed. After each 5-minute session the participants rated their perceived exertion using the Borg scale^15^. Participants then rested for approximately 15 min or until their heart rate returned to baseline. The second compression spring was again randomized and inserted into the manikin, and the same 5-minute CPR sequence and post-session Borg scale rating were repeated. After another 15-minute rest period, the final compression spring was inserted into the manikin. Participants performed the final 5-minute CPR session following the same procedure and again rated perceived exertion using the Borg scale.

### Data collection

The data collected from each participant included demographic information, such as age, gender, height, weight, calculated BMI, resting heart rate and oxygen saturation. CPR efficiency data were also recorded, including overall performance rating, number of compressions per sequence, mean chest compression depth, hand position rating, chest compression adequate depth rating, good chest recoil rating, average chest compression rate, and good chest compressions within the target rate range. The angle of elbow flexion/extension of the dominant arm was collected, as well as post-CPR heart rate and Borg rating of perceived exertion.

### Statistical analysis

Quantitative variable results are presented as mean values ± standard deviation (SD) along with minimum and maximum values. Comparisons between the two groups (male and female) were performed using an unpaired t-test. Comparisons between the three groups (standard spring, soft spring, hard spring) were performed using one-way ANOVA plus Tukey’s post hoc test. The relationships between BMI and CPR efficiency metrics (depth, rate, and recoil), as well as between BMI and elbow flexion/extension data, were analyzed using Pearson’s correlation coefficient. Linear regression analysis was then performed to investigate these relationships further. Multiple linear regression analysis was used to explore the combined influence of BMI, elbow flexion/extension, and CPR efficiency variables (depth, rate, and recoil). SPSS Statistics version 26 software (IBM) was used for the statistical analysis. Statistical significance was set at *p* < 0.05 and *p* < 0.01, as indicated in the tables and figures.

## Results

### Chest compression depth is compromised with increased spring stiffness in female participants compared to males

**Fig. 2:**
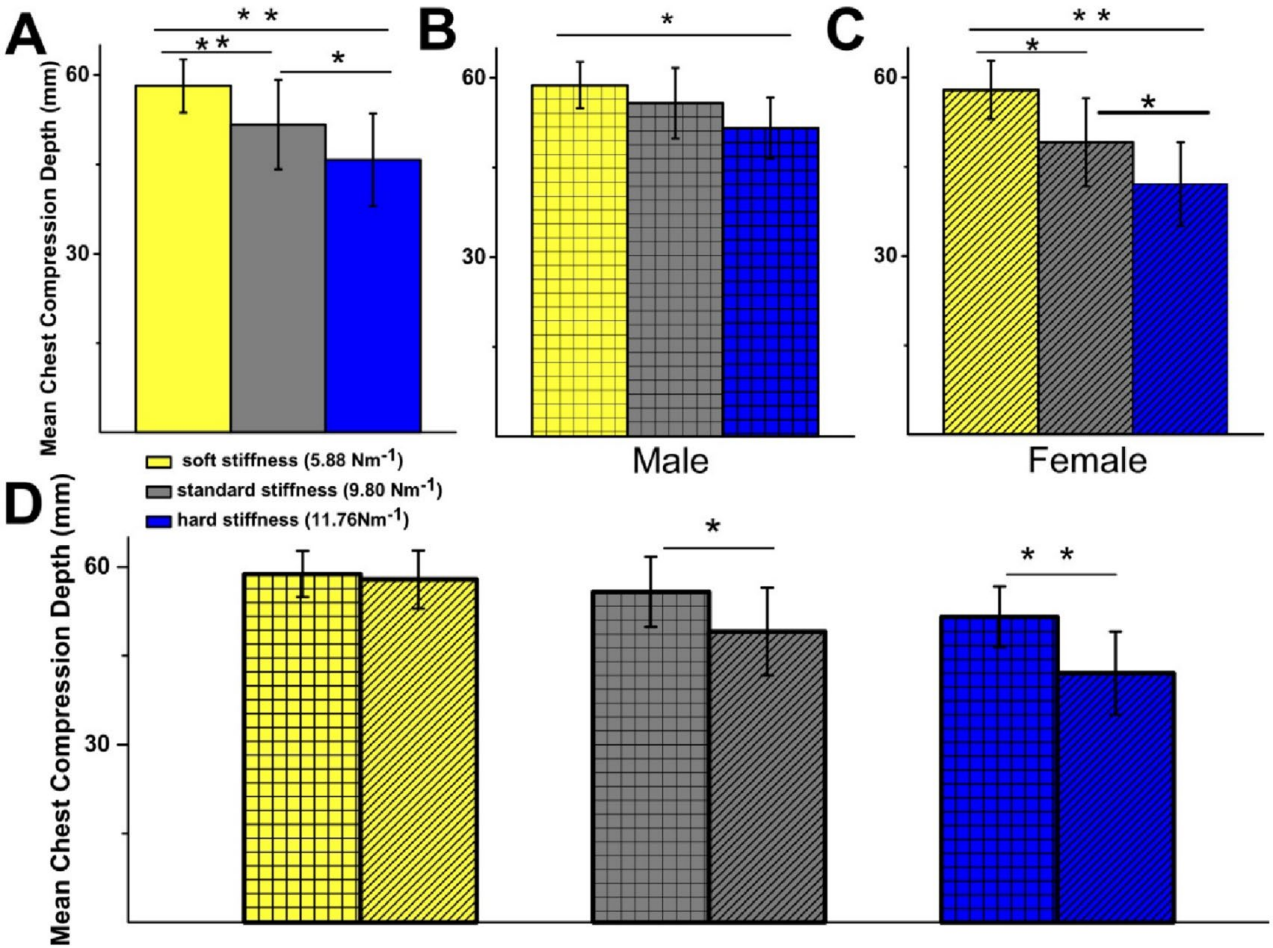
Spring stiffness affects mean chest compression depth (mm) in both males and females. (**A**) Mean chest compression depth for all participants (in mm): soft 58 ± 4, standard 52 ± 8, hard 46 ± 8 mm. Statistically significant difference was observed between soft and hard stiffness (*p* = 0.001), soft and standard stiffness (*p* = 0.006), and standard and hard stiffness (*p* = 0.01). (**B**) Mean chest compression depth for males (in mm): soft 59 ± 4, standard 56 ± 6, hard 52 ± 5. Statistically significant difference was observed between soft and hard springs only (*p* = 0.02). (**C**) Mean chest compression depth for females (in mm): soft 58 ± 5, standard 49 ± 7, hard 42 ± 7. Statistically significant difference was observed between soft and hard stiffness (*p* = 0.001), soft and standard stiffness (*p* = 0.002), and standard and hard stiffness (*p* = 0.02). (**D**) Comparison of mean chest compression depth between male and female participants for 3 springs shows no statistically significant difference for soft spring between males (59 ± 4 mm) and females (58 ± 5 mm). Statistically significant difference was observed for spring of standard stiffness (males 56 ± 6 mm vs. females 49 ± 7 mm, *p* = 0.035) and hard stiffness (males 52 ± 5 mm vs. females 42 ± 7 mm, *p* = 0.002). Male *n* = 9, Female *n* = 14, *Significance at *p* < 0.05. ***Significance at the *p* < 0.01 level.

Overall, chest compression depth was affected by spring stiffness in all participants (Fig. 2A). Mean chest compression depths for all participants were: soft spring, 58 ± 4 mm (SD); standard spring, 52 ± 8 mm (SD); and hard spring, 46 ± 8 mm (SD). Statistically significant differences were observed between the soft and hard springs (58 ± 4 mm vs. 46 ± 8 mm, *p* = 0.001), between the soft and standard springs (58 ± 4 mm vs. 52 ± 8 mm, *p* = 0.006), and between the standard and hard springs (52 ± 8 mm vs. 46 ± 8 mm, *p* = 0.01). However, when the data was analysed with respect to gender, it became clear that for male participants, a statistically significant difference was observed only between the soft and hard springs, with mean chest compressions depths of 58 ± 5 mm and 52 ± 5 mm, respectively (*p* = 0.02) (Fig. 2B).

In contrast, for female participants, statistically significant differences in compression depth were observed between all springs (Fig. 2C). When comparing between genders, statistically significant differences were seen for the normal spring (male: 56 ± 6 mm vs. female: 49 ± 7 mm, *p* = 0.035) and for the hard spring (male: 52 ± 5 mm vs. female: 42 ± 7 mm, *p* = 0.002) (Fig. 2D).

Our data show that both female and male participants demonstrated significant differences in chest compression depth with increasing spring stiffness. Chest compression depth remained within the guideline recommended value of at least 50 mm, except for female participants performing chest compressions on the hard spring.

### Chest compression rate is maintained at recommended levels despite increased spring stiffness

**Fig. 3:**
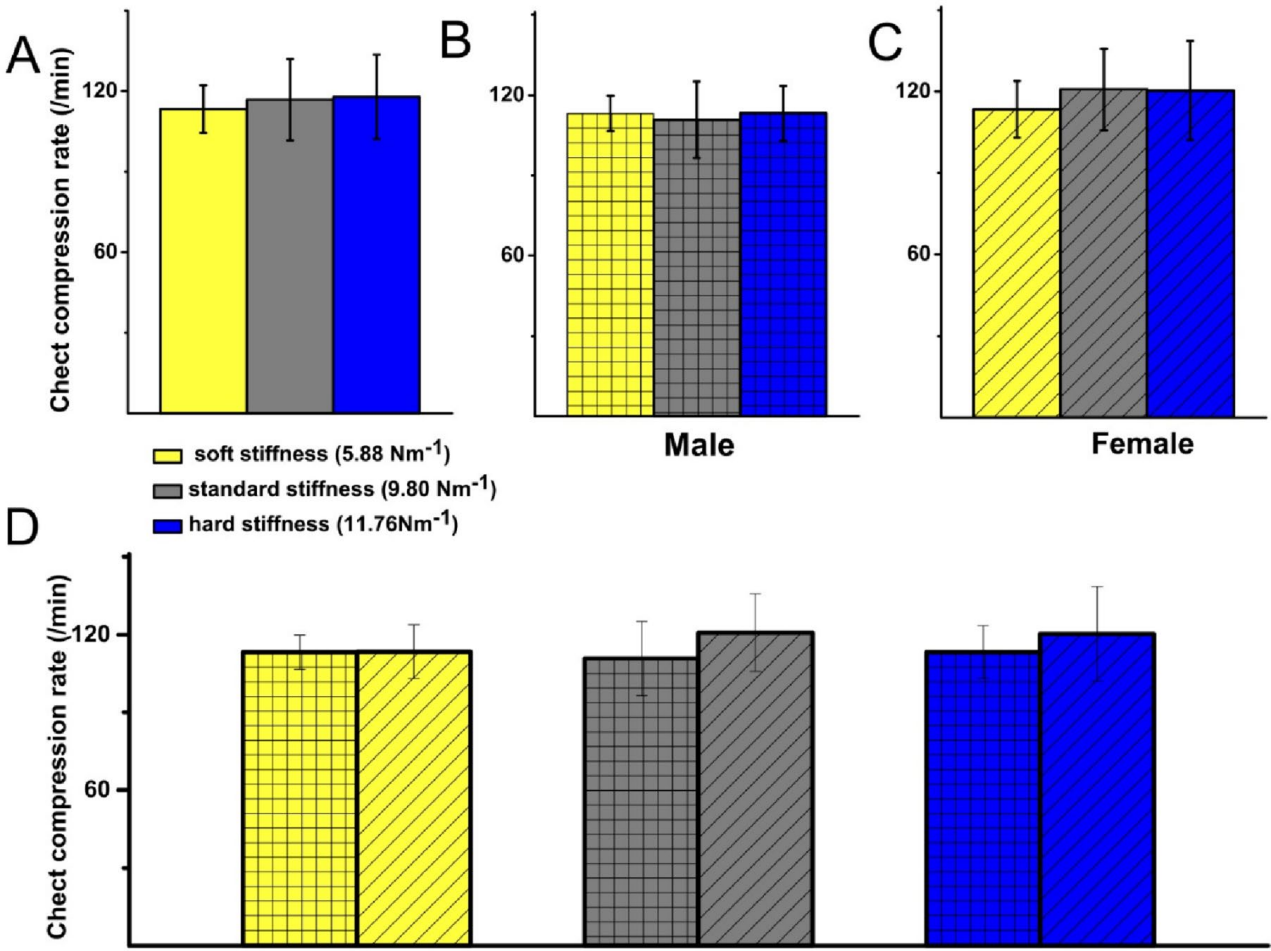
Spring stiffness does not affect mean chest compression rate (/min) in all participants. (**A**) Mean chest compression rate measured for all participants (/min) soft spring: 113 ± 9, standard spring 116 ± 15, hard spring 118 ± 16 mm, showed no statistically significant difference for all participants: soft vs. standard (*p* = 0.65), soft vs. hard (*p* = 0.51), and standard vs. hard (*p* = 0.89).(**B**) Mean chest compression rate for males (/min): soft spring 113 ± 7, standard spring 111 ± 14, hard spring 113 ± 10. No statistically significant difference was observed between soft vs. standard springs (*p* = 0.88), soft vs. hard springs (*p* = 0.89) and standard vs. hard springs (*p* = 0.87). (**C**) Mean chest compression rate for females (/min): soft spring 113 ± 10, standard spring 110 ± 14, and hard spring 121 ± 18. No statistically significant difference found for all participants: soft vs. standard springs (*p* = 0.41), soft vs. hard springs (*p* = 0.39), and standard vs. hard springs (*p* = 0.89). (**D**) Mean comparison of chest compression rate for male vs. female participants for the 3 springs shows no statistically significant difference for soft spring between males (113 ± 7,) and females (113 ± 10, *p* = 0.89). No significance was also observed for standard spring (/min) (males 116 ± 15 vs. females 111 ± 14, *p* = 0.13) and hard spring (males 118 ± 16 vs. females 121 ± 18/min, *p* = 0.27) Male *n* = 9, Female *n* = 14.

We next examined the effect of spring stiffness and gender on chest compression rate (Fig. 3). There was no statistically significant difference in chest compression rate for all participants across the three springs: soft spring, 113 ± 9 compressions/min; standard spring, 116 ± 15 compressions/min; and hard spring, 118 ± 16 mm compressions/min (Fig. 3A). Similarly, no significant differences were found when analysing by gender. For male participants, compression rates were 113 ± 7, 111 ± 14, and 113 ± 10 compressions/min for the soft, standard and hard springs, respectively (Fig. 3B). For female participants, the corresponding values were 113 ± 10, 110 ± 14, and 121 ± 18 compressions/min (Fig. 3C). When comparing male and female participants, chest compression rates were comparable for all spring stiffness conditions (Fig. 3D).

### Elbow flexion increases progressively with spring resistance

Elbow flexion and extension during CPR performance was investigated to determine whether these movements can be used as a compensatory mechanism to maintain adequate chest compression depth and rate with increasing spring stiffness. To assess whether elbow flexion adapted to increasing thoracic stiffness, a Friedman ANOVA was conducted to compare both mean elbow flexion and its standard deviation across the three spring stiffness conditions (soft , standard , and hard) in a repeated-measures design involving 23 participants. The analysis revealed a statistically significant difference in mean elbow flexion across the three conditions (χ²^2^ = 24.61, *p* < 0.00001), with Kendall’s coefficient of concordance indicating moderate within-subject consistency (W = 0.53). Mean elbow flexion increased progressively as spring stiffness increased, with observed values of 4.18° (SD = 2.21) for the soft spring, 5.10° (SD = 3.02) for the standard spring, and 7.38° (SD = 4.60) for the hard spring.

A similar pattern was observed for variability in elbow flexion, which also differed significantly between spring conditions (χ^2^ = 12.52, *p* = 0.0019). However, the within-subject concordance was weaker (W = 0.27). The standard deviation of elbow flexion increased from 1.90° (SD = 0.96) with the soft spring, to 2.24° (SD = 1.08) with the standard l spring, and 2.93° (SD = 1.59) with the hard spring. These results suggest that both the magnitude and variability of elbow flexion increased as spring stiffness intensified, supporting the hypothesis that elbow flexion is used as a compensatory strategy under greater thoracic resistance.

**Fig. 4:**
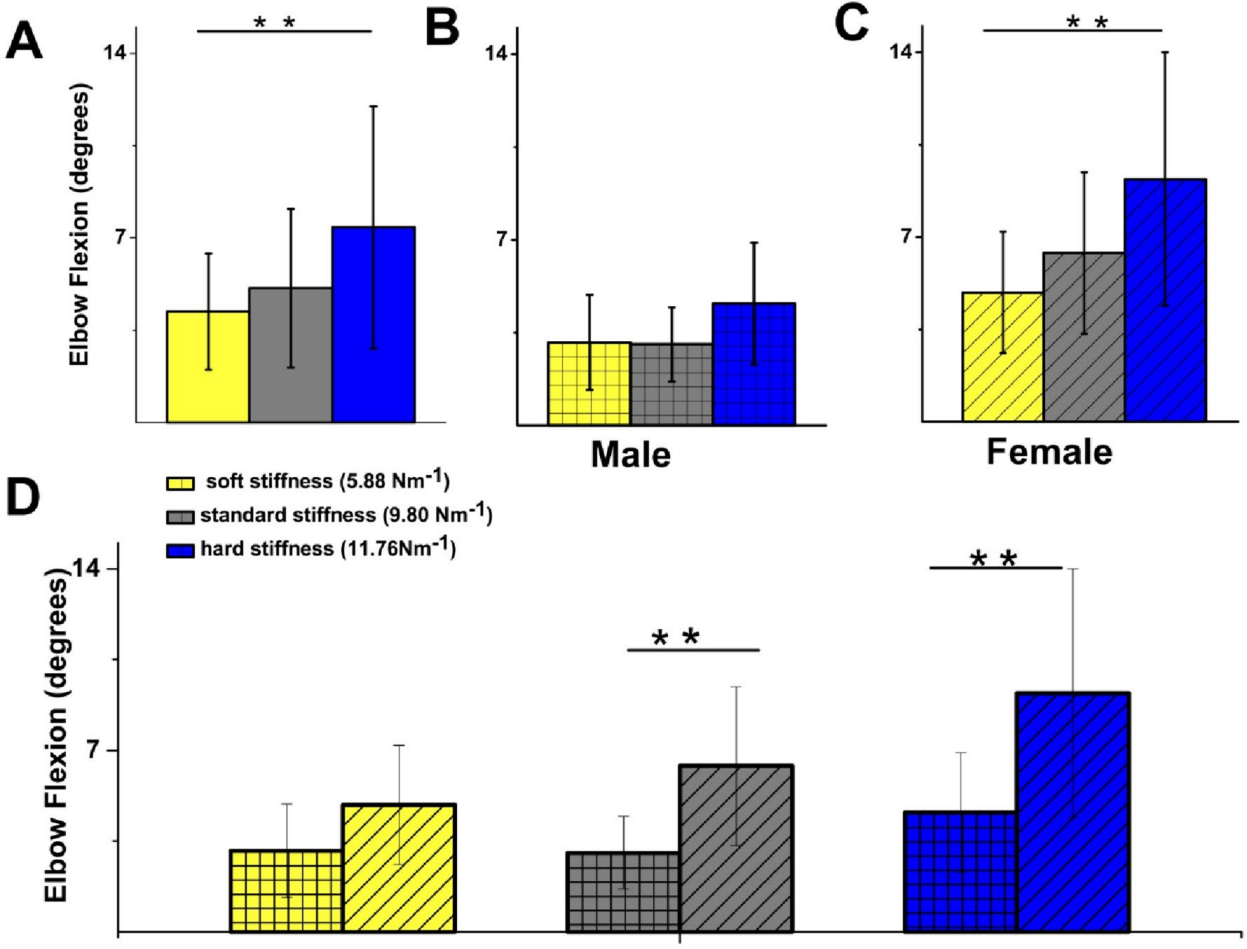
Mean elbow flexion/extension is increased in female participants for standard and hard springs. (**A**) Comparison of mean elbow flexion/extension for all participants for 3 spring stiffnesses (degrees): soft 4 ± 2, standard 5 ± 3, hard 7 ± 6, showed no statistically significant difference for all participants for soft vs. standard (*p* = 0.62), standard vs. hard (*p* = 0.07), but significant for soft vs. hard (*p* = 0.006). (**B**) Comparison of mean elbow flexion/extension for male participants for 3 spring stiffnesses (degrees): soft 3 ± 2, standard 3 ± 1, hard 5 ± 2. No statistically significant difference was observed between soft vs. standard springs (*p* = 0.89), soft vs. hard (*p* = 0.27) and standard vs. hard (*p* = 0.23). (**C**) Comparison of mean elbow flexion/extension for female participants for 3 spring stiffnesses (/min): soft 5 ± 2, standard 6 ± 3, hard 9 ± 5. No statistically significant difference was observed for all participants: soft vs. standard (*p* = 0.48), standard vs. hard (*p* = 0.11), but significant difference for soft vs. hard (*p* = 0.006). (**D**) Comparison of mean elbow flexion/ extension for male vs. female participants for 3 springs shows no statistically significant difference for soft spring (5.88 N/m) between male (3 ± 2,) and female (5 ± 2) participants (*p* = 0.07). Significant difference was observed for springs of standard stiffness (degrees): males 3 ± 1 vs. females 6 ± 3, *p* = 0.006 and hard stiffness: males 5 ± 2 vs. females 9 ± 5, *p* = 0.01). Male *n* = 9, Female *n* = 14 **Significance at *p* < 0.01 level.

Additionally, we aimed to identify any differences between male and female participants (Fig. 4). No statistically significant differences in mean elbow flexion were observed between springs for male participants. In contrast, for female participants, mean elbow flexion/extension differed significantly between the soft and hard springs (*p* = 0.007) (Fig. 4). Moreover, significance differences between males and females were observed for springs of standard stiffness (male: 3 ± 1° vs. female: 6 ± 3°, *p* = 0.006) and hard stiffness (male: 5 ± 2° vs. female 9 ± 5, *p* = 0.01).

### Objective (heart rate) and subjective (Borg scale) exertion after each CPR sequence show significant differences between male and female participants

**Fig. 5:**
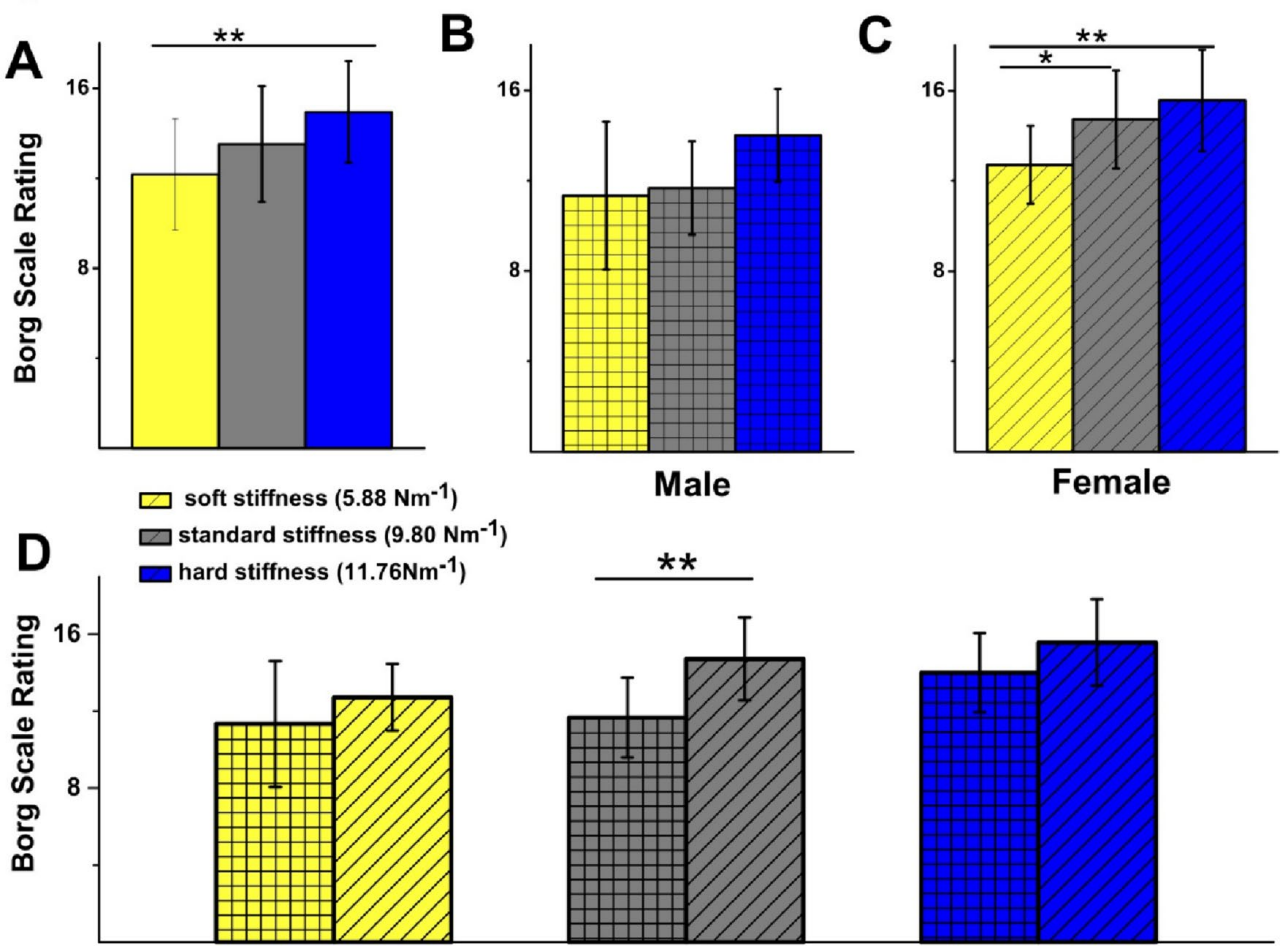
Female participant perceived exertion correlated with spring stiffness. (**A**) Comparison of perceived exertion using Borg scale for all participants for 3 springs: soft 12 ± 2, standard 14 ± 3, hard 15 ± 2. There was no statistically significant difference for all participants for soft vs. standard spring (*p* = 0.16), standard vs. hard spring (*p* = 0.12), but significant for soft vs. hard spring (*p* = 0.001). (**B**) Comparison of perceived exertion using Borg scale for male participants: soft 11 ± 3, standard 12 ± 2, hard 14 ± 2. No statistically significant difference was observed between soft vs. standard springs (*p* = 0.89), soft vs. hard (*p* = 0.09) and standard vs. hard (*p* = 0.15). (**C**) Comparison of perceived exertion using Borg scale for female participants: soft 13 ± 2, standard 14 ± 2, hard 16 ± 2. Females showed statistically significant difference for: soft vs. standard (*p* = 0.03) and soft vs. hard (*p* = 0.002), but showed no significant difference between standard vs. hard (*p* = 0.52). (**D**) Comparison of perceived exertion using Borg scale for male vs. female participants for 3 springs shows there was no statistically significant difference for soft spring (male 12 ± 2 and female 11 ± 3, *p* = 0.20) and for hard spring (male 14 ± 2 vs. female 16 ± 2, *p* = 0.11). Significant difference was observed for standard stiffness (males 12 ± 2 vs. females 14 ± 2, *p* = 0.003). Male *n* = 9, Female *n* = 14, *Significance at *p* < 0.05 level. ***Significance at the *p* < 0.01 level.

Objective and subjective exertion were measured after each CPR performance using the subjective self-rated Borg scale and objective post-CPR heart rate. The aim was to identify whether participants perceived and physiological exertion differed between different springs (Fig. 5A), and between genders (Fig. 5B–D).

While male participants did not show significant differences in perceived exertion between springs (Fig. 5B), female participants demonstrated a statistically significant increase in Borg rating for increased spring stiffness (soft : 13 ± 2 vs. standard : 14 ± 2, *p* = 0.03; and soft: 13 ± 2 vs. hard: 16 ± 2, *p* = 0.002; Fig. 5C). Borg ratings were also significantly different between males and females after CPR performed on the standard spring (male: 12 ± 2 vs. female: 14 ± 2, *p* = 0.003; Fig. 5D), suggesting that females perceived the normal spring to be more demanding than males. However, Borg ratings were similar between genders for easy and hard springs.

**Fig. 6:**
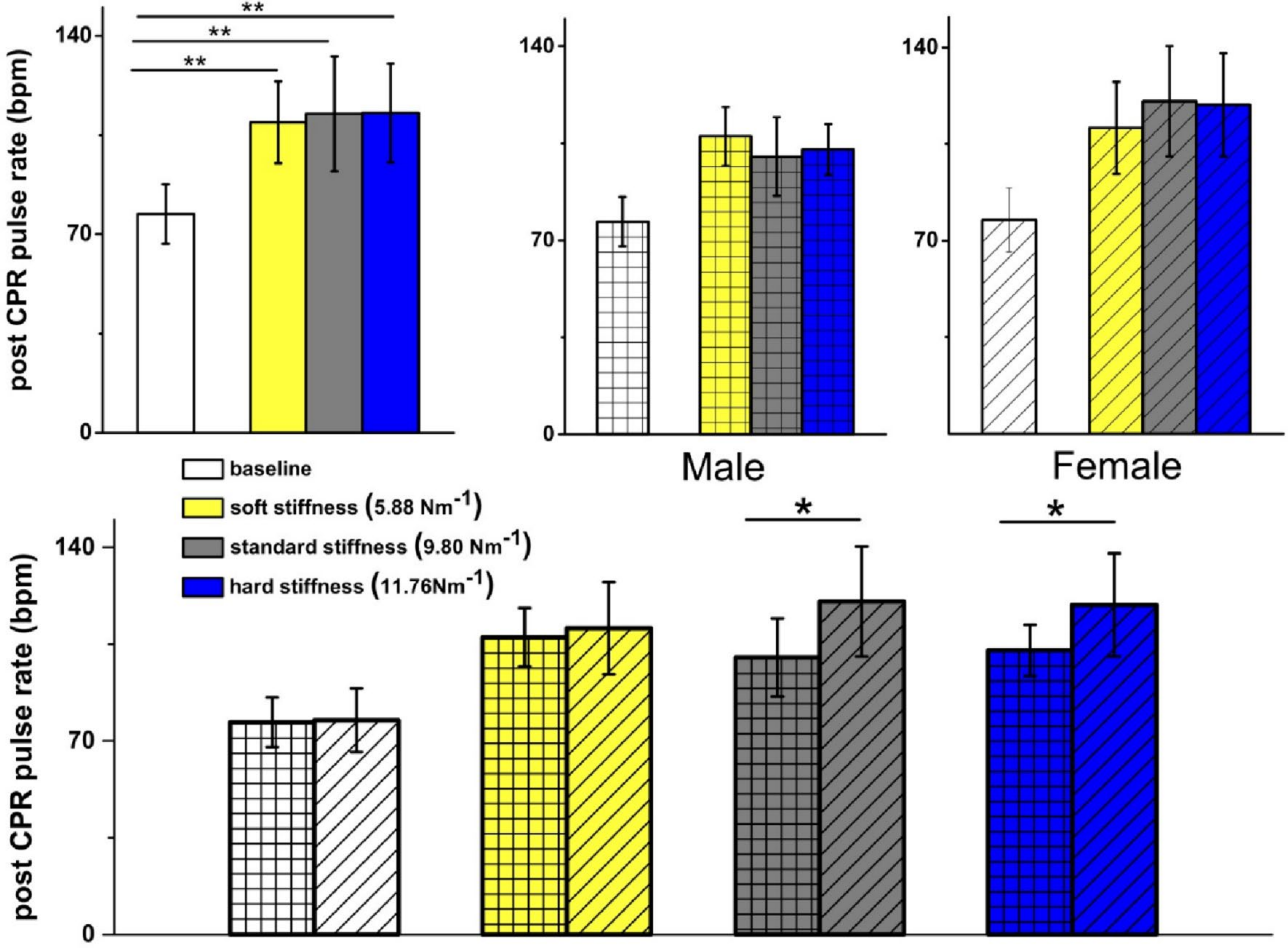
Comparison of pulse rate (beats per minute, bpm) pre- and post-CPR as objective measurement of exertion shows greater exertion for females compared to males using standard and hard springs. (**A**) Comparison of pulse rate (bpm) pre- and post-CPR for all participants for 3 springs: baseline 77 ± 10, soft 110 ± 14, standard 112 ± 20, hard 112 ± 17, showed no statistically significant difference for all participants for soft vs. standard (*p* = 0.90), standard vs. hard (*p* = 0.90), and soft vs. hard (*p* = 0.90). The baseline heart rate increased significantly in all cases (soft *p* = 0.001, standard *p* = 0.001, hard *p* = 0.001). (**B**) Comparison of pulse rate (bpm) pre- and post-CPR for male participants: baseline 77 ± 9, soft 108 ± 11, standard 100 ± 14, hard 103 ± 9. No statistically significant difference was observed between soft vs. standard springs (*p* = 0.50), soft vs. hard (*p* = 0.77) and standard vs. hard (*p* = 0.90). (**C**) Comparison of pulse rate (bpm) pre- and post-CPR for female participants: baseline 76 ± 12, soft 110 ± 17, standard 120 ± 20, and hard 120 ± 19. No statistically significant difference observed for soft vs. standard (*p* = 0.45), soft vs. hard (*p* = 0.56), and standard vs. hard (*p* = 0.90). (**D**) Comparison of pulse rate (bpm) pre- and post-CPR for male vs. female participants for 3 springs shows no statistically significant difference for soft spring between male (108 ± 11) and female (110 ± 17) participants (*p* = 0.62) Significant difference was observed for standard stiffness (male 100 ± 14 vs. female 120 ± 20, *p* = 0.016) and for hard stiffness (male 103 ± 9 vs. female 120 ± 19, *p* = 0.02). Male *n* = 9, Female *n* = 14. *Significance at *p* < 0.05 level. ***Significance at *p* < 0.01 level.

Post-CPR heart rate increased significantly from baseline levels (Fig. 6A) however, the post-CPR heart rate did not differ significantly between spring stiffness conditions for either males or females (Fig. 6B and C). When comparing genders, female participants showed higher post-CPR heart rates for both the standard and hard springs ( standard spring: male 100 ± 14 bpm vs. female 120 ± 20 bpm, *p* = 0.016; hard spring: male 103 ± 9 bpm vs. female 120 ± 19 bpm, *p* = 0.02).

### Significant negative correlation between participants’ BMI and the degree of elbow flexion/extension as a function of spring stiffness

**Fig. 7:**
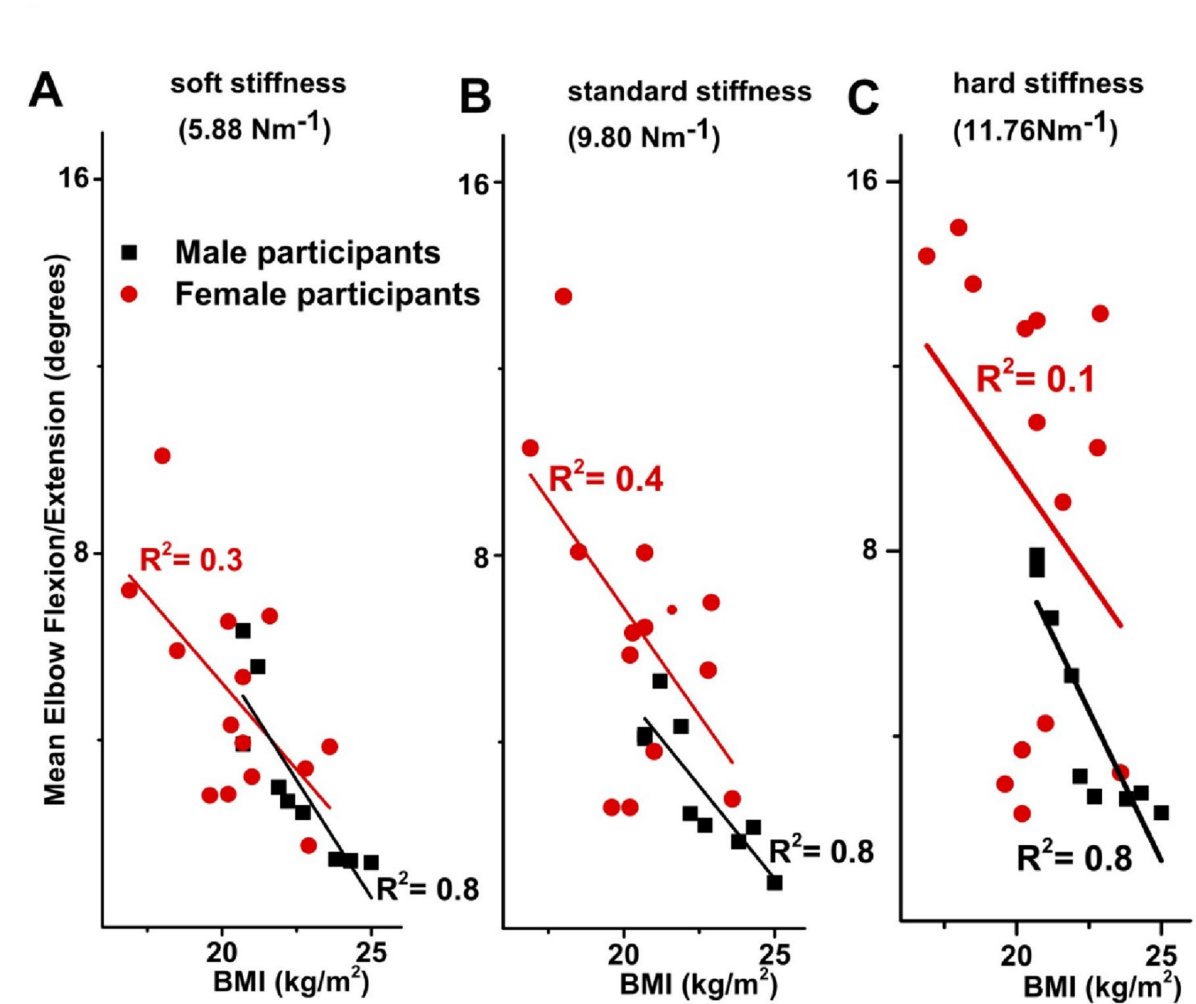
There is significant negative correlation between BMI of male and female participants and the mean elbow flexion/extension during CPR performed on all 3 springs. (**A**) Relationship between the BMI of male and female participants and mean elbow flexion/extension during CPR performed on the spring with soft stiffness (5.88 N/m). Increasing BMI and mean elbow flexion/extension were significantly negatively correlated, both in males (*r* = − 0.872, *p* = 0.001**) and females (*r* = − 0.626, *p* = 0.008**). BMI was also found to significantly predict mean elbow flexion/extension, in both males (*R* = 0.872, B = − 1.004, *p* = 0.002**) and females (*R* = 0.626, B = − 0.741, *p* = 0.017*). *Significance at the *p* < 0.05 level. **Significance at the *p* < 0.01 level. (**B**) Relationship between BMI of male and female participants and the mean elbow flexion/extension during CPR performed on the spring with standard stiffness (9.8 N/m). Increasing BMI and mean elbow flexion/extension were significantly negatively correlated, both in males (*r* = − 0.874, *p* = 0.000**) and females (*r* = − 0.568, *p* = 0.017*). BMI was also found to significantly predict mean elbow flexion/extension, in both males (*R* = 0.874, B = − 0.798, *p* = 0.002**) and females (*R* = 0.568, B = − 0.920, *p* = 0.034*). *Significance at *p* < 0.05 level. **Significance at *p* < 0.01 level. (**C**) Relationship between the BMI of male and female participants and the mean elbow flexion/extension during CPR performed on the spring with hard stiffness (11.76 N/m). Increasing BMI and mean elbow flexion/extension were significantly negatively correlated in males (*r* = − 0.888, *P* = 0.001**). BMI was also found to significantly predict mean elbow flexion/extension in males (*R* = 0.888, B = − 1.298, *p* = 0.001**). **Significance at the *p* < 0.01 level.

Linear correlation analysis was performed to further explore the relationships between BMI and mean elbow flexion/extension for both male and female groups. For male participants, a significant negative correlation between BMI and elbow flexion/extension was observed across all three spring stiffness conditions: standard  (9.8 N/m,*R* = − 0.874, *p* = 0.002), soft  (5.88 N/m, *R* = − 0.872, *p* = 0.002), and hard (11.76 N/m, *R* = − 0.888, *p* = 0.001). For female participants, the linear correlation between BMI and elbow flexion/extension was also negative and significant for both the standard spring (9.8 N/m, *R* = − 0.568, *p* = 0.034) and the soft spring (5.88 N/m, *R* = − 0.626, *p* = 0.017), but not for the hard spring (11.76 N/m, *R* = − 0.353, *p* = 0.216) (Fig. 7).

### Perceived fatigue increased with elbow motion variability without corresponding heart rate changes

To explore whether variations in elbow motion influence fatigue during CPR, two multiple linear regression analyses were conducted on the pooled dataset (*N* = 69), which included measurements from all three spring stiffness conditions. In both models, mean elbow flexion and its standard deviation were entered as independent variables, representing average and variability in elbow positioning, respectively.

In the first model, Borg score was used as the dependent variable to assess perceived fatigue. The model was statistically significant (*F*(2,66) = 13.42, *p* < 0.00001), explaining 27% of the variance in perceived exertion (*R*^2^ = 0.29, adjusted *R*^2^ = 0.27). While mean elbow flexion was not a significant predictor (*p* = 0.14), the standard deviation of elbow flexion emerged as a significant positive predictor (*β* = 0.90, *p* = 0.002), indicating that participants with greater variability in arm movement reported higher perceived fatigue.

In contrast, when post-CPR heart rate was used as an objective proxy for physical fatigue, the regression model did not reach statistical significance (*F*(2,66) = 2.81, *p* > 0.05), and neither predictor showed a significant association. This suggests that although perceived exertion increased with elbow motion variability, this pattern was not mirrored in the physiological response measured via heart rate.

### Predictive value of elbow flexion dynamics for chest compression performance metrics

Finally, multiple linear regression analyses were conducted on the combined dataset including all spring stiffness conditions (*N* = 69), to evaluate whether elbow flexion dynamics—specifically the mean angle and its variability (standard deviation)—predict key indicators of chest compression performance. The five dependent variables were tested: mean chest compression depth (mm), percentage of compressions at adequate depth, average compression rate (compressions/min), percentage of compressions within the recommended rate range, and percentage of compressions with full chest recoil.

For mean chest compression depth, the model was statistically significant (*F*(2,66) = 5.77, *p* = 0.0049), with an R^2^ of 0.15, indicating that elbow kinematics explained 15% of the variance. However, neither mean elbow flexion (*p* = 0.36) nor its variability (*p* = 0.72) were individually significant predictors. A similar pattern was observed for the percentage of compressions at adequate depth. The model was significant (*F*(2,66) = 5.43, *p* = 0.0065; R^2^ = 0.14), yet neither predictor reached statistical significance on its own (*p* = 0.12 and *p* = 0.70, respectively). In contrast, models predicting chest compression rate (*F*(2,66) = 1.41, *p* = 0.252; R^2^ = 0.041), percentage within target rate (*F*(2,66) = 0.38, *p* = 0.688; R^2^ = 0.011), and full chest recoil (*F*(2,66) = 1.39, *p* = 0.256; R^2^ = 0.04) were all non-significant, with minimal variance explained.

Collectively, these results suggest that although elbow flexion dynamics may contribute to some aspects of chest compression quality—particularly depth—they are not robust or consistent predictors across the full spectrum of CPR performance indicators. Given the limited sample size, particularly within subgroups defined by sex and spring stiffness, more detailed modeling within these strata was not feasible. Future studies with larger cohorts should explore whether the predictive value of elbow flexion dynamics varies across specific combinations of participant characteristics and compression resistance levels.

## Discussion

High quality CPR has been shown to increase survival outcomes^16–18^. To our knowledge, this study provides the first terrestrial data demonstrating that elbow flexion and extension, in addition to upper torso acceleration, may contribute to achieving high-quality CPR when the rescuer is smaller than the victim. Rescuer body weight has previously been suggested as a key determinant of effective external chest compressions (ECC) in novice rescuers, with those weighing less than 56 kg being more than six times as likely to produce ECC below that of current guidelines^8,9^.

Our findings suggest that when resistance within the thoracic cage increases, representing a larger patient or one with underlying respiratory disease, smaller rescuers compensate by increasing their elbow flexion angle to generate more force. This adaptation enables them to achieve the recommended compression depth in accordance with current CPR guidelines^2^. Previous studies have utilized varying resistance springs in resuscitation manikins to simulate differing levels of thoracic stiffness^19^. These studies also demonstrated that with increasing thoracic stiffness, the traditional straight-arm CPR technique results in decreased ECC quality and depth, accompanied by greater operator fatigue^19^.

Analysis of mean elbow flexion/extension between male and female participants showed statistically significant differences during CPR performed on the normal and hard springs (Fig. 4), with greater flexion/extension observed in female participants. A significant difference between springs was also found among female participants (Fig. 4).

Pearson’s correlation analysis revealed a statistically significant relationship between BMI and mean elbow flexion/extension (moderate to strong negative correlation) for all three springs in male participants, and for the normal and easy springs in female participants (Fig. 7). Linear regression analysis further demonstrated that BMI significantly predicted mean elbow flexion/extension for all three springs in males, as well as for the standard and soft springs in females (Fig. 7). These results support the hypothesis that smaller rescuers, characterized by lower BMI, may need to increase elbow flexion/extension to maintain CPR quality, particularly to achieve adequate ECC depth.

ECC depth should ideally be more than 50 mm^2,16^. In this study, male participants were able to achieve this target for CPR performed on all three springs (Fig. 2B), despite a decline in average ECC depth from 59 to 52 mm. Female participants achieved this target depth for CPR performed on the soft spring (58 ± 5 mm) and the standard spring (49 ± 7 mm), but not for the hard spring (42 ± 7 mm) (Fig. 2C). Although previous studies have reported a significant relationship between BMI and adequate ECC depth, this study did not demonstrate this relationship^8,9^. One possible explanation is that smaller participants with lower BMI compensated through increased elbow flexion/extension, allowing them to maintain CPR quality despite reduced body weight.

Chest compression rate should ideally be between 100 and 120 compressions per minute^2,16^. Both male and female participants achieved this target for all three springs (Fig. 3B,C). Male participants maintained a consistent compression rate, whereas female participants exhibited a trend towards increasing their rate of ECC as the spring resistance increased, reaching 121 ± 18 compressions per minute with the hard spring, albeit not statistically significant. This could represent a compensation response in which rescuers instinctively increase compression rate as compression depth decreases.

This finding is similar to what occurs during CPR in hypogravity, where there is effectively a reduction in body weight, particularly of the upper body. A recent review of hypogravity CPR recommends that, in reduced gravitational environments, rescuers should flex and extend their upper limbs to generate enough force to perform adequate ECC depth. Rate of ECC is preserved while performing CPR in hypogravity simulations, even in Martian and Moon simulated gravitational fields. There is also a logical pattern, in which as gravitational force decreases (equivalent to a great reduction in rescuer body weight), there is a corresponding increase in arm flexion/extension angle, as well as a higher level of perceived exertion^6,7^.

A similar increase in heart rate between males and females was observed in this study, with no statistically significant difference between the two groups or between spring stiffness conditions (Fig. 6). However, there was a significant increase in Borg scale ratings of perceived exertion in females compared to males (Fig. 5). Female participants reported finding CPR increasingly difficult as spring resistance rose, likely due to their lower average body weight and BMI, making it more difficult to generate the force needed to perform adequate ECCs. This highlights the limitations of using heart rate alone as a single objective measure of work intensity. Performing ECC continually over a five-minute period represents a blend of endurance and strength activity, incorporating both aerobic and anaerobic effort. Therefore, more sophisticated physiological measures are likely needed to accurately measure rescuer performance.

Altering how muscles are used during CPR, whether through fatigue, strength limitations, or suboptimal recruitment of muscle groups, can significantly impair CPR quality. For example^20^, activity in key prime mover muscles such as the deltoids, pectoralis major, biceps, latissimus dorsi, and upper and middle trapezius declines markedly over time during both hands-only and 30:2 CPR, which is associated with reductions in compression depth, rate, and overall force generation. Rescuers with greater upper‐body strength and anaerobic power are better able to maintain proper compression depth and rate, especially during prolonged CPR or repeated resuscitation cycles^21^. Strength training focused on the chest‐compression muscles has been shown to improve compression depth, maintain correct compression fraction, and help preserve performance over time^22^. In summary, altered muscle utilization, resulting from fatigue or insufficient fitness, can lead to a drop in CPR effectiveness, especially in key quality parameters such as compression depth, rate, and full chest recoil.

The findings of our study suggest that where there is a large discrepancy between rescuer and patient size, such as a small rescuer performing CPR on a large patient, the traditional straight-arm CPR technique may need to be modified to comply with current guideline recommendations. This can be done by augmenting upper body acceleration with flexion and extension of the upper limbs to generate extra force to provide the required depth of ECC. This should be taken into consideration when revisiting CPR guidelines on Earth, in order to increase the likelihood of delivering high-quality CPR and possibly improving patient survival, particularly for novice rescuers^8,9^. These results also add to the evidence regarding CPR in hypogravity environments, suggesting there may be a minimum body weight needed to perform CPR effectively using the traditional straight-arm technique. Below this threshold, greater elbow flexion/extension may be needed to produce adequate ECC quality in reduced gravitational fields.

As previously discussed, heart rate alone is a limited indicator of the metabolic demand of exercise, as demonstrated by the lack of significant differences in HR between males and females, despite the variance in perceived exertion on the Borg scale. More sophisticated assessments of anaerobic and aerobic measures of the endurance and strength elements of CPR could be incorporated into future studies to help develop training protocols. This could include oxygen uptake (VO_2_) with spirometry, aerobic capacity testing, one-repetition max and blood lactate level measurements. Another way to build on this study would be to recruit larger sample sizes with a broader range of low, normal, and higher BMI categories, while also considering percentage body fat and total muscle mass. CPR performance could then be correlated with these factors to determine whether, for example, there is a minimum threshold of muscle mass or aerobic fitness level needed to perform effective CPR. This may be particularly relevant for CPR performance in micro and hypogravity environments where physical deconditioning affects multiple systems. Collecting such extra data could help define a minimum body weight, upper-body strength and aerobic capacity needed to perform effective CPR and guide the development of training protocols.

A one-tailed t-test was used in this study based on the hypothesis that individuals with lower BMI would be more challenged when performing CPR on individuals much larger than themselves and would rely more on arm flexion than participants with normal BMI. The study recruited a relatively small sample of participants, including 9 males and 14 females who successfully completed the experiment. This shortfall may have limited the statistical power of the analysis, increasing the risk of Type II error (i.e., failing to detect a true effect). As a result, the findings should be interpreted with caution, as the reduced sample size may affect both the precision and generalizability of the results. Nevertheless, the study still provides valuable preliminary insights and highlights trends that can inform future research with adequately powered samples.

## Conclusion

These findings suggest a potential compensatory mechanism that may help maintain CPR performance compliant with guideline recommendations in situations when the rescuer is significantly smaller than the patient, similar to techniques suggested for delivering CPR in hypogravity environments^6^. Smaller and novice rescuers could benefit from modifying the traditional CPR technique by incorporating flexion and extension of the upper limbs to generate more force, thereby improving compression depth and overall CPR quality. This could positively influence the outcomes of CPR and should be taken into consideration when revising the internationally recognized CPR guidelines. Further research is needed to determine other key factors in performing CPR, such as aerobic capacity, muscle mass, strength and endurance training, as these could have implications for both terrestrial and hypogravity CPR.

## Data Availability

All data is available in the main text and supplementary figures.
